# *FOS* and Other Genes Linked to Oxidative Stress Are Upregulated in Women Suffering from Endometriosis and Might Negatively Impact the Oocyte Developmental Competence During ICSI

**DOI:** 10.3390/antiox15070879

**Published:** 2026-07-16

**Authors:** Pawel Kordowitzki, Joanna Kochan, Jakub Wyroba

**Affiliations:** 1 Department of Basic and Preclinical Sciences, Nicolaus Copernicus University, 87-100 Torun, Poland; 2 Institute of Advanced Studies, Nicolaus Copernicus University, 87-100 Torun, Poland; 3 Department of Gynecology, European Competence Center for Ovarian Cancer, Charité Medical University, 13353 Berlin, Germany; 4 Department of Animal Reproduction, Anatomy and Genomics, University of Agriculture, 31-120 Krakow, Poland; joanna.kochan@urk.edu.pl; 5 Malopolski Institute of Fertility Diagnostics and Treatment–KrakOvi, 31-120 Krakow, Poland; jakub.wyroba@krakovi.med.pl; 6 Fertility Disorders Clinic, Andrzej Frycz Modrzewski Krakow University, 31-120 Krakow, Poland

**Keywords:** aging, oocyte, blastocyst, AMH, intracytoplasmic sperm injection, endometrioma, endometriosis, *c*-*FOS*, *FOS*, *SRF*

## Abstract

Purpose: We aim to elucidate the impact of endometriomas on oocyte developmental competence and identify genetic alterations that may influence ICSI success rates in women of different ages. Methods: In this retrospective study, two groups of patients have been analyzed: 108 women with a history of severe endometriosis (stages III and IV), who underwent OPU and ICSI after endometriosis surgery, and a control group, built out of 108 women with no endometriosis history. The Turku Endometriosis Database was used for differential gene expression analysis. Results: The number of aspirated oocytes (endometrioma group: 2.75 ± 0.32 vs. control 10.32 ± 0.54) was significantly (*p* < 0.0001) lower in patients with endometrioma excision, and the number of generated blastocysts was significantly (*p* < 0.0001) lower when compared to patients without endometriosis (endometrioma group: 1.09 ± 0.13 vs. control group: 3.76 ± 0.23). Additionally, AMH levels were significantly lower (*p* < 0.001) in the endometriosis-affected group below the age of 35 years (1.65 ± 0.15) than in the unaffected control group (2.99 ± 0.27) within the same age group. The mRNA expression of the following genes related to oxidative stress was significantly (*p* < 0.05) upregulated in the endometriosis group compared with healthy controls: *DES*, *P2RX1*, *FOS*, *SRF*, *RASD2*, *SLITRK3*, and *TEAD3*. Gene Ontology and pathway enrichment analyses demonstrated a significant (*p* < 0.0001) enrichment of oxidative stress–associated biological processes and signaling pathways, including responses to reactive oxygen species and hypoxia, HIF-1, MAPK, PI3K–Akt, NF-κB, and apoptotic signaling, supporting oxidative stress as a central molecular mechanism underlying the observed transcriptomic alterations. Conclusions: In conclusion, this retrospective study provides a holistic perspective on the impact of endometrioma and its oxidative stress effect on oocyte developmental rates and ICSI outcomes across different age groups.

## 1. Introduction

Oxidative stress represents a critical impediment to reproductive success, arising from an imbalance between reactive oxygen species (ROS) and cellular antioxidant capacity [1,2]. In oocytes and preimplantation embryos, pathological ROS concentrations trigger lipid peroxidation, DNA fragmentation, and apoptosis, significantly impairing developmental competence and fertilization rates [1,3]. Central to the cellular response to such oxidative insults is the *FOS* gene, a classical ROS-responsive immediate-early gene and a key component of the AP-1 transcription factor complex [4]. FOS expression is highly sensitive to the redox environment, where its timely regulation is vital for oocyte maturation and subsequent embryonic viability [5]. Importantly, maternal age and gynecologic pathologies, such as endometriosis, also affect the balance of oxidative stress in the ovarian microenvironment. Endometriosis is defined by the presence of endometrial-like tissue outside the uterus, affecting approximately one in ten women of reproductive age, and can increase to 30% in infertile women [6]. The disease can manifest in various locations, including the ovaries (endometrioma), the pouch of Douglas, and the vesico-uterine space [7]. In addition, endometriosis can lead to anatomical distortions in the reproductive tract, reduce ovarian reserve, and decrease oocyte and embryo quality, all of which can impact fertility [7,8,9]. Importantly, the significant delay in diagnosis of endometriosis contributes to the overall burden and complexity of managing endometriosis and additionally leads to postponement of motherhood.

Notably, intracytoplasmic sperm injection (ICSI) has become a common therapeutic option for numerous women to achieve a desired pregnancy [10,11,12]. Although ICSI can overcome some oocyte impairments, fertilization rates may still be compromised [11]. Ovarian endometriomas (OE) add another layer of complexity to the ICSI procedure. OEs induce a localized, chronic inflammatory and fibrotic response within the ovarian microenvironment, and surgical excision of OE can negatively affect ovarian architecture and follicular development [5,11,12,13]. Notably, the ovary is remarkably organized, and its parenchyma contains the ovarian follicles. The entire lifetime supply of these crucial ovarian follicles is established long before birth [13]. These follicles develop during fetal life, a process during which oocytes initiate meiosis and then arrest in most mammalian species at prophase I, remaining in this dormant state until puberty begins. In cases of diminished ovarian reserve, synchronizing follicular growth and using GnRH protocols are crucial for improving pregnancy rates [14]. Notably, women with endometriosis still face challenges during ART cycles, needing frequently more than one ART cycle to reach clinical pregnancy [11]. Combination medical and surgical therapies without ART have not been shown to improve fertility outcomes significantly, and such approaches could potentially delay further fertility treatments and postpone motherhood to a more advanced age [15,16]. Some researchers suggest that endometrioma may induce inflammation and oxidative stress in the ovarian cortex, a condition also characteristic of ovarian aging, and potentially alter follicular and oocyte development [10].

Therefore, the aim of this retrospective study was to investigate the impact of endometriosis (stages III-IV) and endometrioma excision on AMH levels, ovarian reserve, oocyte developmental competence, and ICSI outcomes in women of different ages, using a GnRH agonist and antagonist protocol. We further aimed to identify genetic markers linked to oxidative stress in women with endometriosis that could affect oocyte developmental competence and embryo implantation success. We hypothesize that the oxidative stress in the follicular microenvironment of patients with endometriosis will lead to the upregulation of genes related to oxidative stress, such as *FOS*, which thereby impacts the quality of oocytes.

## 2. Methods

### 2.1. Study Design

In this retrospective study, 216 women who underwent only intracytoplasmic sperm injection (ICSI) cycles at a Krakovi Clinic in Kraków (Poland) from 2021 to 2024, were analyzed. In all these 216 ICSI cases, no paternal factors for infertility were noted, to avoid a potential confounding factor by spermatozoa. The ICSI procedure is predominantly chosen in this clinic, as previously described [17]; therefore, conventional in vitro fertilization (IVF) has not been considered. We have analyzed two groups of patients: 108 women with a history of severe endometriosis (stages III and IV) and endometrioma excision, who underwent ICSI, and a control group, built out of 108 women with no endometriosis history. The visual quality and morphology of the oocyte and blastocyst were assessed in a blinded manner by one embryologist. The patients’ age ranges were evenly distributed across both groups, with 26 to 44 years in the endometriosis group and 28 to 43 years in the control group. Besides endometriosis, other gynaecologic disorders, such as adenomyosis and endometritis, have not been presented in the patients’ history, and cases with male factor of infertility have not been taken into consideration.

### 2.2. Ovarian-Sparing Laparoscopic Cystectomy Using Cold Dissection and Minimal-Hemostasis Strategy

All procedures were performed by an experienced minimally invasive gynecologic surgeon using a standardized fertility-preserving laparoscopic technique. The primary surgical objective was complete excision of the ovarian cyst while minimizing injury to the surrounding healthy ovarian cortex and preserving ovarian reserve. Following the establishment of pneumoperitoneum (12–14 mmHg CO_2_) and the placement of a standard four-port laparoscopic setup, the pelvis was systematically inspected, and adhesiolysis was performed as required using sharp dissection. Dense adhesions involving the ovary were released using cold laparoscopic scissors whenever feasible to avoid collateral thermal injury. The ovarian cortex overlying the cyst was opened with cold laparoscopic scissors using a linear cortical incision positioned along the antimesenteric border of the ovary. No monopolar or bipolar electrosurgery was used during the cortical incision. Gentle, blunt, and sharp dissection was then employed to identify the natural cleavage plane between the cyst capsule and the normal ovarian cortex. The cyst wall was removed using the stripping technique by applying gentle countertraction between two atraumatic laparoscopic graspers. Constant traction–countertraction was maintained while progressively separating the capsule from the normal ovarian tissue with meticulous blunt dissection. Areas of increased tissue adherence were carefully released using cold laparoscopic scissors rather than energy devices to minimize inadvertent excision of healthy ovarian cortex. After complete excision, the ovarian bed was carefully inspected. Hemostasis was primarily achieved by allowing spontaneous physiological coagulation under temporary reduction in intra-abdominal pressure to approximately 6–8 mmHg for several minutes. During this observation period, gentle compression was applied only when necessary using a moistened atraumatic laparoscopic gauze or blunt instrument for 2–5 min over focal bleeding sites. Routine electrocoagulation was deliberately avoided. If persistent capillary oozing remained after spontaneous hemostasis and compression, additional mechanical compression was repeated. Thermal coagulation was reserved exclusively for clinically significant bleeding that could not be controlled by conservative measures and, if required, was limited to isolated pinpoint applications using the lowest effective bipolar power setting (<20 W) and the shortest possible activation time (<1 s). No monopolar coagulation was used for ovarian hemostasis. The ovarian edges were left unsutured unless extensive cortical separation or persistent bleeding necessitated approximation. When suturing was required, a fine absorbable monofilament suture (4-0 poliglecaprone or equivalent) was placed using interrupted intracorporeal stitches, taking care to avoid excessive tissue tension or cortical inversion.

### 2.3. Identification of Differentially Expressed Genes Related to Oxidative Stress

For this analysis, the Turku Endometriosis Database was used, which contains results generated from endometrial tissue samples obtained from patients diagnosed with endometriosis (*n* = 345) and age-matched healthy controls (*n* = 63), following informed consent and institutional review board approval. Total RNA was extracted from tissue samples and subjected to microarray-based gene expression profiling. Gene expression levels were quantified and normalized across samples. To avoid potential bias, only data from the Turku Database that were generated during the early proliferative phase of the menstrual cycle have been used, as this stage has the lowest impact on the oxidative stress level of the endometrium and helps ensure a comparable hormonal status. Only patients without local inflammation have been considered. The candidate genes included in the present study were selected based on a comprehensive review of the literature on their established roles in oxidative stress, redox homeostasis, mitochondrial function, DNA damage repair, and ovarian aging. Particular emphasis was placed on genes previously implicated in the regulation of reactive oxygen species (ROS), antioxidant defense mechanisms, cellular senescence, follicular depletion, and age-related decline in ovarian function. Given the central role of oxidative stress in ovarian aging and its contribution to diminished ovarian reserve and impaired oocyte quality, the selected gene panel was designed to encompass key molecular pathways linking oxidative damage with ovarian physiology. Thus, the investigated biomarkers represent biologically plausible candidates with demonstrated relevance to both oxidative stress-mediated cellular processes and ovarian aging, providing a strong mechanistic rationale for their inclusion in the present study. To identify genes whose expression levels differ between endometriosis patients and healthy controls, we performed differential expression analysis using Welch’s *t*-test for each gene. This method was chosen to account for unequal variances and sample sizes between groups. For each gene, we calculated: (1) mean expression in diseased and healthy groups, (2) log_2_ fold change (log_2_FC) defined as log_2_[(diseased mean + 0.001)/(healthy mean + 0.001)], (3) t-statistic and *p*-value using two-tailed Welch’s *t*-test, and (4) effect size (Cohen’s d) to quantify the magnitude of difference. Multiple testing correction was applied using the Benjamini–Hochberg false discovery rate (FDR) method, with significance defined as FDR < 0.05. A total of 73 genes were identified as significantly differentially expressed (31 upregulated, 42 downregulated in endometriosis).

### 2.4. Gene Ontology Enrichment Analysis

Protein-coding genes from the top 10 oxidative-associated genes were subjected to gene ontology (GO) enrichment analysis to identify overrepresented biological processes. Non-coding RNAs (including microRNAs and small nuclear RNAs) were excluded from this analysis. GO enrichment was performed using g: Profiler (version 2024) with the following parameters: organism = Homo sapiens, sources = GO Biological Process (GO:BP), significance threshold method = FDR, user threshold = 0.05. GO terms with a minimum of 2 genes and FDR < 0.05 were considered significantly enriched.

### 2.5. Statistical Analysis of Clinical Data

Non-parametric data were analyzed using the Mann–Whitney or Kruskal–Wallis test, followed by a Dunn’s multiple-comparisons test, as we did not assume a specific distribution for the variables measured on nominal or ordinal scales. Other tests used for the single analyses are mentioned in the respective figure legends. Differences were considered significant when the *p*-value was ≤0.05. The statistical analysis was performed using the R software, version 4.4.1 (R Project for Statistical Computing).

## 3. Results

### 3.1. Impact of Endometriosis on Ovarian Follicles and Developmental Competence

**Figure 1:**
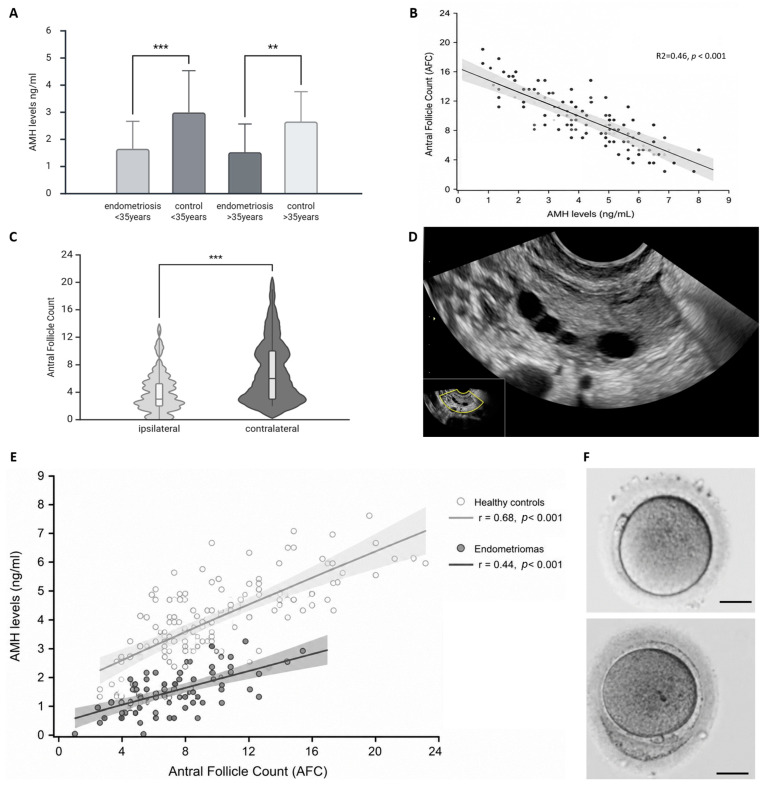
Anti-Müllerian-Hormone levels and antral follicle counts in endometriosis patients and controls. Panel (**A**) shows the AMH levels of patients undergoing ICSI in two different age groups, below and above 35 years, with and without endometriosis (controls). Panel (**B**) shows Linear regression analysis demonstrating the relationship between serum anti-Müllerian hormone (AMH) levels and antral follicle count (AFC). Each point represents an individual patient. The solid regression line represents the fitted linear regression model, and the shaded area indicates the 95% confidence interval. The regression model estimates the expected change in AFC associated with a 1 ng/mL decrease in serum AMH, demonstrating a significant decline in AFC with decreasing AMH concentrations. The regression equation, coefficient of determination (R^2^), and corresponding *p*-value are displayed within the figure. Panel (**C**) shows the antral follicle coun for the ipsilateral (endometrioma) and contralateral ovary (unaffected ovary). Panel (**D**) shows an exemplary ultrasound image of an ovary used to analyze the AFC and perform OPU. Panel (**E**) shows the correlation between serum anti-Müllerian hormone (AMH) levels and antral follicle count (AFC) in women with ovarian endometriomas and healthy controls. Scatter plots illustrate the relationship between serum AMH concentrations (ng/mL) and AFC in women diagnosed with ovarian endometriomas and healthy controls. Solid lines represent the fitted linear regression models for each group, with shaded areas indicating the 95% confidence intervals. A significant positive correlation between AMH levels and AFC was observed in both groups; however, women with endometriomas consistently exhibited lower AMH concentrations and reduced AFC compared with healthy controls across the entire range of ovarian reserve values. Correlation coefficients (Spearman’s r) and corresponding *p*-values are shown within the figure. Panel (**F**) shows two representative microscopic photographs of metaphase II oocytes: the upper one from an unaffected patient and the lower one from a patient with an endometrioma, showing irregular ooplasma and vacuoles. Scale bar indicates 30 μm. ** stands for *p* < 0.01, *** stands for *p* < 0.001.

To investigate the extent to which endometriomas alter follicular development and reduce oocyte developmental competence, we first analyzed antral follicle count (AFC) and AMH blood concentrations in patients of different maternal ages. Our analysis revealed that AMH levels were significantly (*p* < 0.001) lower in the endometriosis-affected group aged 35 years or less (1.65 ± 0.15) compared to the unaffected control group (2.99 ± 0.27) within the same age group (Figure 1). In patients older than 35 years, AMH levels are also significantly (*p* < 0.01) lower in the endometriosis-affected group (1.52 ± 0.2) than in unaffected counterparts (2.66 ± 0.21). To clarify whether laparoscopic surgery, regardless of maternal age, impacts follicular development in the ovary where the endometrioma was removed, we compared the AFC of the ovary with no endometrioma (contralateral) with the AFC of the ovary after endometrioma excision (ipsilateral). As shown in Figure 1, significantly (*p* < 0.001) fewer follicles (3.89 ± 0.36) could be counted at the ipsilateral ovary than at the contralateral ovary (6.93 ± 0.6). This demonstrates that a comprehensive understanding of the physiopathology of endometriomas is pivotal and, therefore, integral to customizing fertility treatment plans to align with the biological variations in each patient. The aforementioned surgery to remove endometriotic lesions is recommended to improve fertility and to increase the chances of conceiving naturally before patients choose ICSI. Moreover, a significant (*p* < 0.001) positive correlation between AMH levels and AFC was observed in both groups (Figure 1), and the downward shift in the regression line in the endometrioma group demonstrates the detrimental effect of endometriomas on ovarian reserve, indicating that, for a given AFC, affected patients generally present with lower circulating AMH levels than healthy women. The negative impact of endometriomas on the morphological quality of metaphase II oocytes is shown in a representative microscopic photograph (Figure 1).

### 3.2. Time Trying to Conceive in Patients with Endometriosis

**Figure 2:**
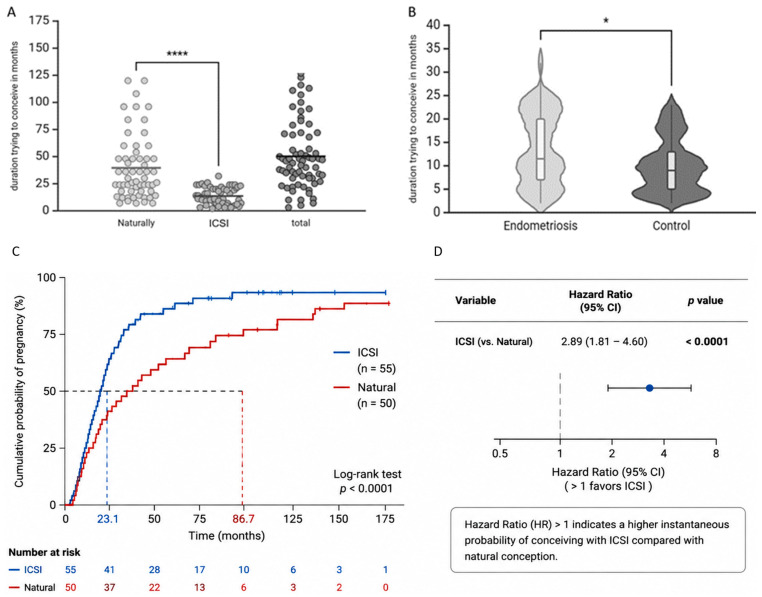
Influence of endometrioma surgery on patients’ fertility and duration of trying to conceive. Panel (**A**) shows the duration of trying to conceive in ICSI patients after endometriosis surgery. Here, the duration in months is depicted and refers to either natural attempts or ICSI. This graph shows that patients after the endometriosis surgery try for a long time to conceive naturally (without success) prior to choosing ICSI. Panel (**B**) shows the duration of trying to conceive with the help of ICSI in the endometriosis and control groups. Here, the duration in months is shown and refers only to ICSI attempts in the endometriosis and control groups. This graph shows that patients after the endometriosis surgery require more time to conceive with the help of ICSI than their unaffected counterparts. Panel (**C**) shows the time-to-pregnancy analysis in women with endometriosis undergoing natural conception or intracytoplasmic sperm injection. Kaplan–Meier curves with Cox proportional hazards regression analysis comparing cumulative pregnancy rates between women with endometriosis attempting natural conception and those undergoing ICSI. The *y*-axis represents the cumulative probability of achieving pregnancy, whereas the *x*-axis denotes time to conception (months). Tick marks indicate censored observations. The solid lines represent the estimated cumulative pregnancy functions for each group. Panel (**D**) shows Hazard ratios (HRs) with corresponding 95% confidence intervals (CIs) were calculated using Cox proportional hazards regression to quantify the relative likelihood of conception over time. * stands for *p* < 0.05, and **** stands for *p* < 0.0001.

**Figure 3:**
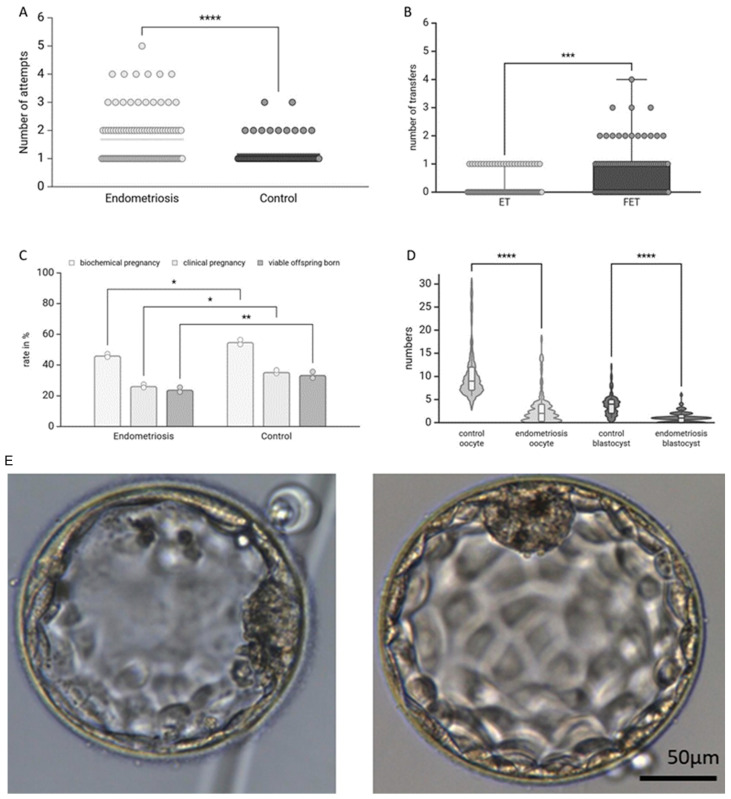
Number of attempts, embryo transfers per patient undergoing ICSI in the endometriosis and control group. Panel (**A**) shows the number of attempts in the endometriosis and control groups. This graph depicts that patients after the endometriosis surgery require more ICSI cycles/attempts to conceive than their unaffected counterparts. Panel (**B**) shows the number and types of embryo transfer in the endometriosis patients. Only a few fresh embryo transfers (ET) have been performed in endometriosis patients, and frozen embryo transfer (FET) has been performed more frequently. Panel (**C**) shows the pregnancy rates for endometriosis patients and respective controls after ICSI. Here, the rates of biochemical pregnancies, clinical pregnancies, and live-born offspring are shown for the two analyzed groups. Panel (**D**) shows the numbers of oocytes and blastocysts generated only from women of advanced age (above 35 years) with (*n* = 28) and without endometriosis (*n* = 32). Panel (**E**), the microscopic photograph on the left shows an exemplary poor quality blastocyst generated from a patient in the endometriosis group, whereas photograph on the right shows an excellent quality blastocyst of the control group. * stands for *p* < 0.05, ** stands for *p* < 0.01, *** stands for *p* < 0.001, and **** stands for *p* < 0.0001.

As can be seen in Figure 2, we have compared the duration of trying to conceive naturally in patients after endometriosis surgery with the duration of trying to conceive with the help of ICSI, after natural attempts. This was also done to underline the psychological stress to which endometriosis patients are exposed when desiring children. The patients enrolled in this study conceived significantly (*p* < 0.0001) faster (13.64 ± 1.02 months) when undergoing ICSI than compared to their natural attempts (39.59± 3.85 months) prior to the decision for ICSI (Figure 2). However, regarding the mean time needed to conceive with the help of ICSI in patients without endometriosis, the duration was significantly (*p* < 0.01) lower (10 ± 0.74) compared to patients with endometriosis (13.64 ± 1.02) (Figure 3). Moreover, patients with endometriosis required significantly (*p* < 0.0001) more ICSI attempts (1.6 ± 0.09) compared to their counterparts without endometriosis (1.15 ± 0.05) (Figure 2). The time-to-pregnancy analysis for women with endometriosis undergoing natural conception or ICSI, along with Kaplan–Meier curves and Cox proportional hazards regression, shows that women suffering from endometriosis undergoing ICSI had a significantly (*p* < 0.0001) higher probability of achieving pregnancy and a shorter time to conception compared with women attempting natural conception (Figure 2). The Cox regression analysis further confirmed that assisted reproductive treatment was independently associated with an increased pregnancy rate after adjustment for relevant clinical covariates. These findings indicate that ICSI substantially shortens the time to pregnancy in women with endometriosis compared with spontaneous conception, highlighting its clinical benefit in overcoming endometriosis-associated subfertility.

### 3.3. Success Rate of ICSI and Embryo Transfer

Notably, in the endometriosis patient group, embryo transfer was performed significantly more often (*p* < 0.001) with vitrified/thawed blastocysts (frozen embryo transfer/FET) than with fresh blastocysts (ET) (Figure 3). This indicates that preparing an endometriosis patient for embryo transfer is significantly more challenging and that blastocysts are often vitrified prior to transfer due to the endometrium not being ready for implantation. In contrast, as shown in Figure 3, the ET of freshly generated blastocysts on day 5 was more frequently performed in the control group (2.09 ± 0.22) than in the endometriosis patient group (0.24 ± 0.04), indicating that the unaffected patients’ uteri were more often well-prepared for direct ET. There was a significantly (*p* < 0.05) higher percentage of poor quality blastocysts (61%) in the endometriosis patients compared to the control group (41%). Microscopic photographs of exemplary blastocysts, both of good and poor quality, are shown in Figure 3. Finally, the ICSI success and pregnancy rates in all groups were analyzed with regard to biochemical pregnancy rates, clinical pregnancy rates, and live birth rates. As can be noticed in Figure 3, all the aforementioned rates were significantly lower in the endometriosis group when compared to unaffected patients; in other words, abortion was more frequent in the endometriosis group. Notably, the oocyte and blastocyst numbers of patients with advanced maternal age were analyzed to investigate how maternal age and endometriosis specifically influence the aforementioned rates, as this remains elusive in the literature. Notably, advanced maternal age (>35 years) not only the number of aspirated oocytes (endometriosis group: 2.75 ± 0.32 vs. control 10.32 ± 0.54) was significantly (*p* < 0.0001) lower in patients with endometriosis, but also the number of generated blastocysts was significantly (*p* < 0.0001) lower when compared to patients without endometriosis (endometriosis group: 1.09 ± 0.13 vs. control group: 3.76 ± 0.23) (Figure 3D).

### 3.4. Differently Expressed Genes Related to Oxidative Stress in Endometriosis and Healthy Patients

Using the Endometriosis Database Turku, the top ten differentially expressed genes have been identified by comparing endometriosis samples with unaffected controls. Among these genes were *DES*, *P2RX1*, *FOS*, *SRF*, *RASD2*, *SLITRK3*, *TBC1D22A*, *TEAD3*, *CRACR2B*, and *ANAPC11*. Interestingly, the mRNA expression of the following genes: *DES*, *P2RX1*, *FOS*, *SRF*, *RASD2*, *SLITRK3*, and *TEAD3* was significantly upregulated (*p* < 0.05) in the endometriosis group compared with healthy controls. The magnitude of upregulation appears particularly pronounced for *FOS*, an immediate-early transcription factor and a classic ROS-responsive gene, suggesting robust transcriptional activation in endometriosis. In contrast, *CRACR2B* and *ANAPC11* display significantly higher expression in healthy samples. Overall, the distribution patterns show consistent shifts in gene expression across the two tested conditions, with limited overlap among transcripts, supporting their potential utility as discriminative molecular markers.

**Figure 4:**
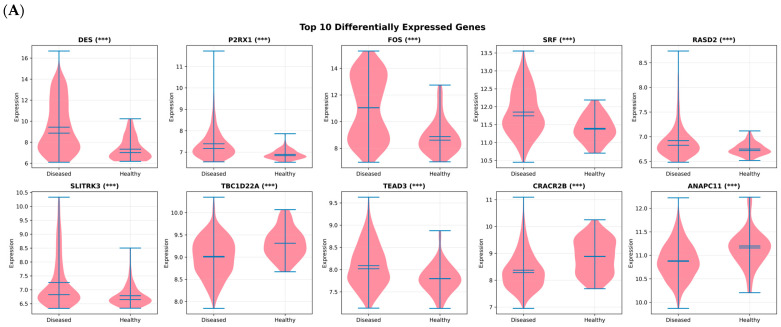

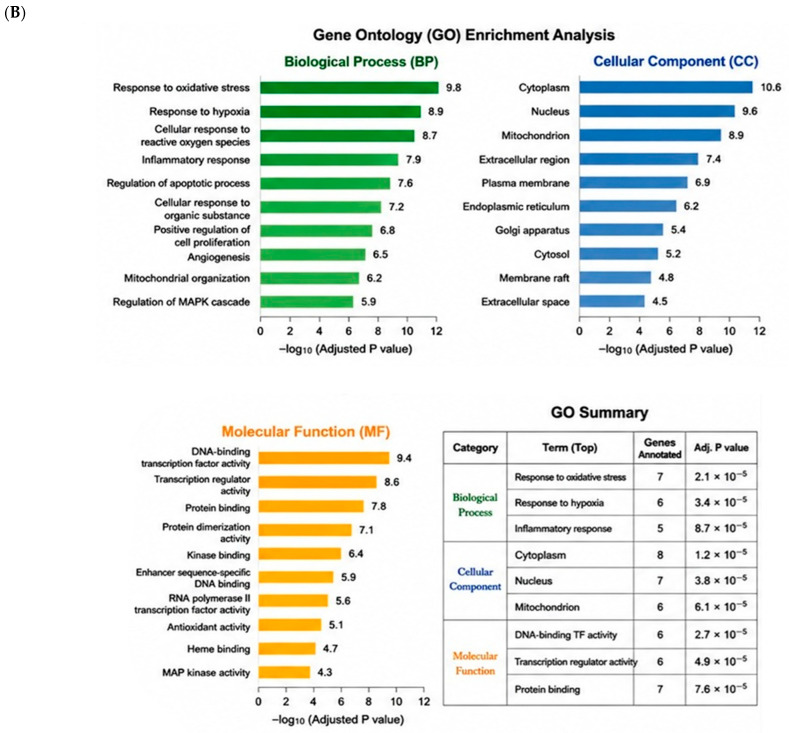

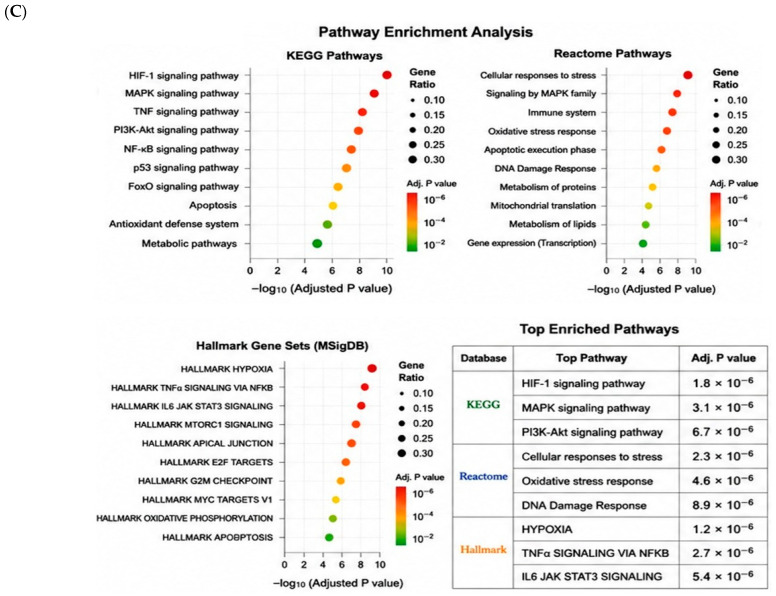
Differential expression of the top 10 genes between endometriosis and healthy tissues. Panel (**A**) shows violin plots which depict normalized gene expression levels for *DES*, *P2RX1*, *FOS*, *SRF*, *RASD2*, *SLITRK3*, *TBC1D22A*, *TEAD3*, *CRACR2B*, and *ANAPC11* in diseased versus healthy samples. The width of each violin represents the density distribution of expression values; horizontal bars indicate central tendency. Statistical significance was assessed using appropriate differential expression analysis). Panel (**B**) shows Gene Ontology (GO) enrichment analysis of the top 10 differentially expressed genes. Enrichment was performed across the three principal GO domains: Biological Process (BP), Cellular Component (CC), and Molecular Function (MF). The bar plots display the most significantly enriched GO terms ranked according to the −log10 adjusted *p*-value. The accompanying summary table lists the representative GO terms together with the number of annotated genes and corresponding false discovery rate (FDR)-adjusted *p*-values. Panel (**C**) shows pathway enrichment analysis of the top 10 differentially expressed genes using the KEGG, Reactome, and MSigDB Hallmark databases. Dot plots illustrate the most significantly enriched pathways ranked according to statistical significance (−log10 adjusted *p*-value). Dot size represents the gene ratio, whereas dot color indicates the FDR-adjusted *p*-value. The summary table highlights the most significantly enriched pathways across all databases. *** stands for *p* < 0.001.

To further investigate the biological significance of the identified differentially expressed genes, comprehensive Gene Ontology (GO) and pathway enrichment analyses were performed (Figure 4). GO enrichment analysis demonstrated that the top differentially expressed genes were predominantly associated with biological processes involved in oxidative stress response, ROS signaling, hypoxia, inflammatory responses, regulation of apoptosis, mitochondrial organization, and MAPK cascade activation. Cellular component analysis indicated significant enrichment within the cytoplasm, nucleus, mitochondria, plasma membrane, and extracellular compartments, whereas molecular function analysis highlighted DNA-binding transcription factor activity, transcriptional regulation, protein binding, kinase activity, antioxidant activity, and heme binding.

Consistent with these observations, pathway enrichment analyses using KEGG, Reactome, and Hallmark databases revealed significant enrichment of pathways involved in HIF-1 signaling, MAPK signaling, PI3K–Akt signaling, NF-κB signaling, TNF signaling, p53 signaling, oxidative stress response, DNA damage response, mitochondrial function, apoptosis, oxidative phosphorylation, inflammatory signaling, and metabolic reprogramming (Figure 4). Notably, Hallmark analysis demonstrated strong enrichment of Hypoxia, TNFα signaling via NF-κB, IL6–JAK–STAT3 signaling, and Oxidative Phosphorylation, further supporting the biological relevance of oxidative stress-related molecular alterations.

## 4. Discussion

Our study provides new genetic and clinical insights and is unique in that we have analyzed oocyte developmental competence and ICSI success in relation to endometriosis and endometrioma excision, not only in terms of live birth rates but also in terms of the time to conception, whether natural or with ICSI. The investigation of genes related to oxidative stress further strengthened this study. This is of outstanding importance for numerous clinicians and patients globally, since the chronic exposure to chronic oxidative stress upon endometriosis in patients undergoing ART is often neglected and requires further elucidation [16].

Therefore, we investigated the impact of endometriomas on reproductive outcomes, specifically focusing on follicular development, oocyte developmental competence, and pregnancy rates, and analyzed the mRNA expression of genes related to oxidative stress.

Our results demonstrate a significant reduction in the number of oocytes and blastocysts generated after surgery for severe endometriosis (stages III and IV) compared with patients without endometriosis. This finding is further corroborated by significantly lower AMH levels in the patient group, who have suffered from endometriosis and all underwent respective surgery. These observations align with existing literature that frequently reports diminished fertility and ovarian reserve in women with endometriosis [17,18], often indicated by reduced AMH levels and lower antral follicle counts [19,20]. The detrimental effects of aged ovaries and endometriomas on ovarian function are multifaceted, potentially involving chronic inflammation, oxidative stress, and direct damage to ovarian tissue, which can impair follicular development and oocyte quality [13,21]. The oxidative stress and inflammatory milieu associated with endometriomas may compromise the microenvironment essential for normal folliculogenesis, resulting in reduced yields of mature oocytes and, consequently, lower blastocyst formation rates, regardless of maternal age [21,22].

This oxidative insult directly damages the oocyte’s cellular components, including its membrane, cytoplasm, and nuclear material, ultimately leading to mitochondrial dysfunction, reduced adenosine triphosphate production, and spindle abnormalities [1,23]. Such compromised oocyte integrity in women is directly correlated with diminished fertilization rates and impaired early embryonic development [23]. Furthermore, the pervasive oxidative stress can extend its deleterious effects to sperm function, contributing to cumulative gamete dysfunction and ultimately, fertilization failure.

A critical finding of our study is the significantly lower AFC from the ipsilateral ovary (where endometrioma was excised) compared to the contralateral ovary. This suggests a potential additional impact of surgical removal of endometriomas on ovarian follicular reserve, which is already diminished due to maternal aging. In this regard, it is worth mentioning that an increasing body of evidence suggests that localized steroidogenesis and progesterone resistance of endometriotic lesions negatively influence follicular development [24,25,26].

The cyst fluid of ovarian endometriomas is rich in ROS and inflammatory mediators, diffuses into adjacent ovarian tissues and follicular fluid, creating a hostile environment that impedes normal follicular development and oocyte maturation [27]. This could explain our findings of a significantly lower AFC in the ovary where the OE was excised, compared to the unaffected contralateral ovary. Furthermore, the mechanical distortion and fibrosis of the ovarian cortex due to OEs can directly hamper follicular growth, since surgical interventions for endometriomas, while often necessary, bear the inherent risk of further compromising ovarian reserve, thereby compounding the fertility challenges in this specific patient population. This is also relevant in the context of maternal aging [28]. Some studies indicate that aggressive endometrioma surgery, especially involving cystectomy, can inadvertently remove healthy ovarian tissue, leading to a reduction in the follicular pool [14,29,30].

Our study observed that while patients conceived significantly faster with ICSI after unsuccessfully attempting to conceive naturally post-endometriosis surgery, those with endometriosis required a longer time to conceive and needed significantly more ICSI attempts overall when compared to the control group. These results align with previous research, which has reported lower success rates for ICSI [31,32,33,34]. The latter-mentioned results underscore the persistent reproductive challenges faced by advanced maternal age and endometriosis [35]. The increased number of ICSI cycles in aged women suffering from endometriosis suggests underlying issues beyond oocyte quantity, such as impaired oocyte quality, reduced embryo developmental potential, or, most notably, compromised endometrial receptivity in endometriosis [36,37,38].

Moreover, our analysis revealed significant differences in biochemical pregnancy rate, clinical pregnancy rate, and viable born offspring rate between endometriosis and unaffected patients, with a higher frequency of abortion in the endometriosis group. This finding is consistent with literature indicating that endometriosis is associated with adverse pregnancy outcomes, including increased rates of miscarriage, ectopic pregnancy, and preterm birth, especially in women of advanced maternal age [39,40]. The mechanisms underlying these complications during reproductive aging are thought to involve systemic inflammatory responses, altered immune regulation, and endometrial dysfunction, which can disrupt early pregnancy development and maintenance. Successful implantation requires a receptive endometrium, a functional embryo at the blastocyst stage of development, and synchronized communication between maternal and embryonic tissues [41,42]. As shown in our analysis, in women with endometriosis, the expression of genes related to oxidative stress, such as *FOS*, *P2RX1*, *SRF RASD2*, and *DES* was significantly altered, reflecting the proliferative and fibrotic characteristics of endometriosis. Overall, the GO and pathway enrichment analyses indicate that the identified differentially expressed genes are not isolated molecular events but constitute an interconnected network involved in redox homeostasis, hypoxic adaptation, inflammatory activation, mitochondrial dysfunction, cellular survival, and metabolic remodeling. These findings provide additional mechanistic support for the hypothesis that oxidative stress-associated signaling pathways from endometriomas seem to contribute to impaired oocyte quality and reduced developmental competence in women with endometriosis.

Interestingly, *FOS* (*c-Fos*) is consistently upregulated in both eutopic and ectopic endometria, acting as a critical early-response gene that drives estrogen-mediated cell proliferation and survival [43,44]. The *FOS* gene encodes a key AP-1 transcription factor subunit [4] that plays a critical role in oocyte maturation and ovulation. Within the ovarian context, *FOS* serves as a critical transcriptional integrator linking gonadotropin signaling to downstream ovulatory and maturational events, with earlier reports confirming the presence of *FOS* transcripts in both germinal vesicle and metaphase II oocytes [45]. The expression dynamics of *FOS* during oocyte maturation reveal a tightly regulated pattern. In Graafian follicles, intense nuclear and cytoplasmic accumulation of *c-Fos* protein has been documented in maturing oocytes at the time of germinal vesicle breakdown, approximately six hours after human chorionic gonadotropin (hCG) administration in gonadotropin-primed mice [46]. Subsequent studies in porcine oocytes have demonstrated that *FOS* mRNA levels decline rapidly and dramatically during the first hour of oocyte–cumulus complex culture under *in vitro* maturation (IVM) conditions [45]. Notably, comprehensive transcriptomic analyses of porcine oocytes revealed that *FOS* exhibited the most pronounced inhibitory effect of any gene across all ontological categories analyzed following IVM, underscoring its pivotal role as a temporally restricted regulator.

In light of our findings of lower oocyte developmental competence in patients with endometriosis, it appears that elevated ROS levels can induce *FOS* expression during follicular rupture, luteolysis, and endometriosis-associated inflammation, positioning the *FOS* gene as a highly plausible oxidative stress marker in ovarian tissue. Interestingly, female *FOS*-null mice fail to ovulate even when administered exogenous gonadotropins, indicating that ovarian FOS expression is critical for successful ovulation [47]. Granulosa cell-specific conditional knockout of FOS (gcFosKO mice) significantly reduces the number of oocytes released upon super-ovulation, accompanied by decreased expression of essential ovulatory genes, including Pgr, Ptgs2, Ptgs1, and Edn2 [47]. Chromatin immunoprecipitation sequencing has identified 1965 *FOS*-binding genes in granulosa cells three hours after hCG administration, demonstrating the breadth of *FOS* transcriptional control over the ovulatory process [47]. *FOS* fulfills these functions, at least in part, by driving prostaglandin biosynthesis in granulosa cells, a process that is essential for cumulus expansion and oocyte release [47]. Importantly, homozygous c-Fos mutant mice display markedly diminished placental and fetal weights, significant perinatal loss of viability, and delayed or absent gametogenesis, indicating that c-Fos contributes to the development and function of multiple reproductive tissues [48]. Beyond *FOS*, the genes *P2RX1*, *SRF*, and *RASD2* are critical mediators of the oxidative stress response. *P2RX1* functions as a purinergic receptor whose activity is modulated by reactive oxygen species, facilitating calcium signaling in response to stress-induced ATP release [49]. The Serum Response Factor (SRF) is a redox-sensitive transcription factor that orchestrates the expression of immediate-early genes to adapt to oxidative environments [50]. Furthermore, *RASD2* integrates metabolic and oxidative signaling pathways, particularly in the context of cellular aging and meiotic maturation [51]. Together, these genes form an essential molecular network that maintains redox homeostasis under pathological conditions, such as endometriomas.

Notably, our reported alterations in endometrial oxidative stress-related gene expression may reflect broader pathophysiological mechanisms contributing to impaired follicular development, reduced oocyte competence, and diminished fertility. *FOS* itself is sensitive to the redox state of the cellular environment. In bovine granulosa cells, oxidative stress induces *FOS* expression, and FOS-mediated apoptosis has been documented following exposure to mycotoxins such as zearalenone [52]. This recent study demonstrated that oxidative stress-triggered transcription factors in granulosa cells, including *FOS* as an AP-1 family member, are differentially regulated by the NRF2 signaling pathway, establishing a critical cross-talk between the antioxidant response master switch and *FOS* [52]. Nevertheless, the present findings should be interpreted with caution, as gene expression was assessed exclusively in endometrial tissue and cannot be assumed to directly mirror molecular alterations occurring within ovarian follicles or granulosa cells. Furthermore, local uterine factors that may substantially influence endometrial oxidative stress, including the composition of the uterine microbiome and subclinical endometritis, were not evaluated in the present investigation. It is also worth mentioning that *FOS* is only one component of a broader network of oxidative stress- and inflammation-associated molecular alterations identified by our transcriptomic analyses.

These factors may independently modulate oxidative stress pathways and gene expression profiles, thereby representing potential confounding variables. Future prospective studies integrating paired ovarian and endometrial molecular profiling, assessment of inflammatory mediators, uterine microbial composition, and longitudinal reproductive outcomes will be essential to elucidate the mechanistic interplay between oxidative stress, ovarian reserve, endometrial receptivity, and reproductive success in women with endometriosis. Moreover, exogenous hormone treatments also significantly influence gene expression in endometrial tissue. Therapy with GnRH agonists, progesterone, or hormonal contraceptives alters the transcriptomic profiles of the endometrium, artificially regulating steroid-responsive pathways and receptors [53]. In our study, we analyzed samples from patients who were not actively taking hormonal medications at the time of tissue collection. Another potential biological confounding factor that can impact genes related to oxidative stress is the patient’s age and body mass index. Therefore, the Turku Endometriosis Database was used in addition to our retrospective clinical analysis, as it allows selection of uniform age groups in the control and study cohorts.

## 5. Conclusions and Limitations

In conclusion, this retrospective study offers a holistic perspective on endometriosis and endometrioma excision, underscoring that these factors substantially compromise ovarian reserve and reproductive success, frequently mandating the application of more intensive and individualized assisted reproductive technologies. The synergistic interplay of chronic inflammation, elevated oxidative stress, and alterations in the genes *FOS* and *DES* within the follicular microenvironment in endometriosis patients diminishes oocyte developmental capacity, fertilization potential, subsequent embryonic development, and implantation. All in all, in our investigation, we aimed to stimulate new and interesting research; therefore, it should be seen as hypothesis-generating rather than mechanistic evidence. Several limitations of the present study should be acknowledged. First, although the clinical validation cohort was derived from a Polish population and the transcriptomic datasets originated from the Turku Endometriosis database, both populations are predominantly of Caucasian ancestry. Nevertheless, potential differences in geographic origin, patient management, treatment strategies, healthcare systems, and environmental exposures may have introduced a degree of heterogeneity that could influence gene expression profiles and biomarker performance. Second, the retrospective nature of both the clinical cohort and the publicly available Turku Endometriosis transcriptomic datasets precludes establishing direct causal relationships between differential gene expression and clinical outcomes. Consequently, the identified biomarkers and molecular pathways should be interpreted as robust associations rather than definitive mechanistic drivers of disease progression. Another limitation is the absence of sperm DNA fragmentation and oxidative stress tests, and therefore these paternal factors cannot be excluded as contributors to blastocyst development and reproductive outcomes. Prospective clinical studies and functional experimental investigations will be essential to confirm the biological significance. Further research is needed to translate our new clinical and genetic insights into targeted therapeutic strategies that improve gamete and embryo quality and enhance reproductive outcomes for women affected by endometriosis.

## Data Availability

Data will be made available on request.
